# Proline Biosynthesis Enzyme Genes Confer Salt Tolerance to Switchgrass (*Panicum virgatum* L.) in Cooperation With Polyamines Metabolism

**DOI:** 10.3389/fpls.2020.00046

**Published:** 2020-02-14

**Authors:** Cong Guan, Xin Cui, Hua-yue Liu, Xue Li, Meng-qi Li, Yun-wei Zhang

**Affiliations:** ^1^ College of Grassland Science and Technology, China Agricultural University, Beijing, China; ^2^ Beijing Key Laboratory for Grassland Science, China Agricultural University, Beijing, China; ^3^ National Energy R&D Center for Biomass (NECB), Beijing, China; ^4^ Beijing Sure Academy of Biosciences, Beijing, China

**Keywords:** switchgrass, *PvP5CS1* and *PvP5CS2*, proline, polyamines, salt stress

## Abstract

Understanding the regulation of proline metabolism necessitates the suppression of two *Δ_1_-pyrroline-5-carboxylate* synthetase enzyme (*P5CS*) genes performed in switchgrass (*Panicum virgatum* L.). The results reveal that overexpressing *PvP5CS1* and *PvP5CS2* increased salt tolerance. Additionally, transcript levels of spermidine (Spd) and spermine (Spm) synthesis and metabolism related genes were upregulated in *PvP5CS OE*-transgenic plants and downregulated in the *PvP5CS RNAi* transformants. According to salt stress assay and the measurement of transcript levels of Polyamines (PAs) metabolism-related genes, *P5CS* enzyme may not only be the key regulator of proline biosynthesis in switchgrass, but it may also indirectly affect the entire subset of pathway for ornithine to proline or to putrescine (Put). Furthermore, application of proline prompted expression levels of Spd and Spm synthesis and metabolism-related genes in both *PvP5CS*-*RNAi* and WT plants, but transcript levels were even lower in *PvP5CS*-*RNAi* compared to WT plants under salt stress condition. These results suggested that exogenous proline could accelerate polyamines metabolisms under salt stress. Nevertheless, the enzymes involved in this process and the potential functions remain poorly understood. Thus, the aim of this study is to reveal how proline functions with PAs metabolism under salt stress in switchgrass.

## Introduction

Plants frequently encounter various environmental biotic and abiotic stresses (i.e., pathogen infection, herbivore attack, drought, salt, cold, heat and toxic metals) (Zhu, 2016). Salt stress seriously limits plant development and productivity leading to both osmotic and ionic toxicity effects on cells and, eventually, disorganized membranes, non-functional proteins, and accumulation of reactive oxygen species (ROS) (Krasensky and Jonak, 2012; Zhu, 2016). ROS cause serious damage through peroxidation of membrane lipid components. These effects on cells can be mitigated by an active oxygen scavenger (e.g., proline, polyamines, glutathione, etc.) and enzymatic protectors (i.e., superoxide dismutase [SOD], catalase [CAT], ascorbate peroxidase [APX] and glutathione peroxidase [GPX]). SODs act as the first line of defense against ROS while CAT subsequently detoxifies H_2_O_2_ (Apel and Hirt, 2004; Krasensky and Jonak, 2012).

In plants, Polyamines (PAs) mainly exist in their free form playing essential roles in cellular function. The putrescine (Put), spermidine (Spd) and spermine (Spm) are the three major forms of polyamines. Putrescine is the central product of the common PAs biosynthetic pathway. It contains two amino groups and is a synthetic precursor of Spd and Spm. Spd and Spm are produced by the sequential addition of aminopropyl moieties to the putrescine skeleton through enzymatic reactions catalyzed by Spd and Spm synthases (SPDS and SPMS). The donor of the aminopropyl groups is decarboxylated S-adenosyl-methionine synthesized from S-adenosyl-methionine by S-adenosyl-methionine decarboxylase (SAMDC). PAs are catabolized by diamine oxidases (DAOs) and polyamine oxidases (PAOs) (Hanfrey et al., 2001; Shao et al., 2012). PAs play important roles in protecting plants against abiotic stress, such as osmotic adjustment, stabilizing membranes to prevent electrolyte leakage and maintaining normal concentrations of reactive oxygen species (ROS) (Bouchereau et al., 1999; Alcázar et al., 2010; Pál et al., 2015; Chen et al., 2018). On the one hand, ectopic expression of SPDS in *Arabidopsis thaliana* resulted a high concentration of Spd and plants showed resistance to salt, drought and cold stress (Kasukabe et al., 2004). On the other hand, overexpression of carnation SAMDC genes produced more amounts of PAs which increased abiotic stresses in *Nicotiana tabacum* (Wi et al., 2006). PA accumulation is coupled with an increase in the activity of PA metabolism genes. For instance, under normal condition, transgenic plants overexpressed with Ethylene responsive factor (*MfERF1*) induced proline enrichment associated with *P5CS* transcripts which was upregulated compared to those of wild type (Zhuo et al., 2018). Additionally, exogenous PAs treatments could increase proline content under osmotic stress (Pál et al., 2018).

Proline, acting as excellent osmolyte, antioxidative defense and signaling molecule, plays a highly beneficial role under environmental stresses (Hayat et al., 2012).When plants are exposed to stressful conditions, accumulated proline functions in maintaining cell osmotic balance, stabilizes membranes to prevent electrolyte leakage and serves as an antioxidant to regulate the level reactive oxygen species (ROS) (Delauney and Verma, 1993). Additionally, proline plays an important role in plant growth and development (Szabados and Savoure, 2010), particularly in the function of cell cycle transition in maize (Wang et al., 2014). Proline contents are determined by its biosynthesis, catabolism and transport. In plants, proline is generally synthesized through the glutamate pathway during osmotic stress. However, ornithine pathway serves as the other pathway to proline synthesis (Hu et al., 1992). In glutamate pathway, proline is produced from glutamate by *Δ_1_-pyrroline-* *5-carboxylate* synthetase (*P5CS*) and *Δ_1_-pyrroline-5-carboxylate* reductase (*P5CR*) enzymes (Hu et al., 1992). In the ornithine pathway, ornithine is transmitted by ornithine-delta-aminotransferase (OAT) producing GSA and P5C which is, thereafter, converted to proline (Roosens et al., 1998). Proline catabolism occurs in mitochondria when proline dehydrogenase (PDH) and P5C dehydrogenase (P5CDH) convert proline to glutamate (Verslues and Sharma, 2010). Intercellular transport of proline occurs in different cellular organelles as exhibited by the compartmentalization of proline metabolism. Proline biosynthesis enzymes (i.e., *P5CS1*, *P5CS2*, and *P5CR*) are predicted to be localized in the cytoplasmic whereas the proline catabolism enzymes (i.e., *ProDH1*, *ProDH2*, *P5CDH*, and *OAT*) are predicted to be localized in the mitochondria (Szabados and Savoure, 2010). In *Arabidopsis*, a *p5cs1* mutant showed hypersensitivity to salt stress. Additionally, *p5cs2* mutations cause embryo abortion during the latter stages of seed development. Thus, P5CS enzymes perform non-redundant functions (Székely et al., 2008). However, the transcriptional pattern of *P5CS* family genes varies among different species. For example, in *Brassica napus*, *Phaseolus vulgaris* and *Oryza sativa*, two *P5CS* family genes were induced by different stresses (Hur et al., 2004; Xue et al., 2009; Chen et al., 2013; Sripinyowanich et al., 2013). Furthermore, *MtP5CS3*, the third gene in *Medicago truncatula*, similar to the other two *MtP5CS* genes, also plays a crucial role in regulating proline accumulation under salinity stress (Kim and Nam, 2013). However, in switchgrass, the extent and functional status of *P5CS* family genes is not clear. Switchgrass (*Panicum virgatum* L.) is a perennial, warm season C4 model grass native to North America recognized as a dedicated bioenergy crop with significant tolerance to abiotic stresses (e.g., heat, cold, and draught) (Casler et al., 2007; Keshwani and Cheng, 2009). This research determined that the functional identification of two *P5CS* genes from *Panicum virgatum*, designated as *PvP5CS1* and *PvP5CS2*, ectopically expressed lines in switchgrass and described their phenotypic and physiological characterization.

## Materials and Methods

### Plant Material

Switchgrass (Alamo, lowland-type, 2n = 4× = 36) was used as wild type for expression profiling and transformation of *PvP5CS1* (Pavir.J02344.1) and *PvP5CS2* (Pavir.J06546.1). Plants were grown in a greenhouse under either a 16h light or 8h dark photoperiod at 25 ± 2°C. According to the description of Hardin, developmental stages of switchgrass are divided into six elongation stages (i.e., E0, E1, E2, E3, E4, and E5) and three reproductive stages (i.e., R1, R2, and R3) (Hardin et al., 2013). At the first stage of elongation, there in none internode, so it is defined as E0. When switchgrass grows the internode, E1 to E5 are defined by the number of internodes present. At the beginning of the reproductive stage, flag leaf comes out, and it is defined as R1. When switchgrass has the fully emerged spikelets without peduncle, it arrived at R2 stage. The R3 stage is defined by fully emerged spikelets and visible peduncle. Plant height, tiller number, leaf length and internode diameter were measured at R1 stage. Each line had three biological replicates, and three tillers were measured in each biological replicate. The harvested above-ground tissues were dried in an oven at 80°C for 72h.

### Bioinformatics Analysis

Based on the amino acid sequence of *AtP5CS1* (NM_001202786.1) and *AtP5CS2* (NM_115419.5), the researchers BLAST-searched the corresponding genes in the Switchgrass Genome Annotation Program (https://phytozome.jgi.doe.gov/pz/portal.html) and found the full-length cDNA sequences of switchgrass *PvP5CS1* and *PvP5CS2*. For the phylogenetic analysis, full-length sequences of P5CS genes from different monocotyledon and dicotyledon plants were retrieved from NCBI (https://www.ncbi.nlm.nih.gov/), the amino acid sequences of two P5CS genes from switchgrass and all those other species, were used to input in the software MEGA 5.0 to do the phylogenetic analysis. DNAMAN software was used to do a homology analysis of two PvP5CS genes. Their full-length cDNA sequences were used to do multiple sequence alignment, and the result was outputted in a form of EMF file. To further analyze the conserved domains of two PvP5CS genes, the researcher inputted their amino acid sequences into the website of NCBI Conserved Domain Search (https://www.ncbi.nlm.nih.gov/Structure/cdd/wrpsb.cgi?) and gained the results of conserved domains.

### Construction of Expression Vectors and Transformation in Switchgrass

For overexpression transformants, cDNA sequences of *PvP5CS1* and *PvP5CS2* were amplified, were fused to binary vector Ubi1301 under *ZmUbi1* promoter. To knockdown both *PvP5CS1* and *PvP5CS2*, an RNAi fragment was amplified by PCR from the conserved regions of *PvP5CS1* and *PvP5CS2* and cloned into *pVT1629* vector (Xu et al., 2011). The recombinant binary vector were introduced into the embryogenic callus (Liu et al., 2015). Wild type (WT) is the control plants. All primers are provided under **Table S1**.

### P5CS and ProDH Activity Assay

*P5CS* activity was measured following the protocol of the *P5CS* ELISA Kit (Suzhou Comin Biotechnology Co. Ltd, China). For calibration purpose standard gradient was measured at 450 nm using a spectrophotometer to make a standard curve. Stop solution was used to change the color from blue to yellow and the intensity of the color was measured. ProDH activity was measured following the protocol prescribed by the kit (Suzhou Comin Biotechnology Co. Ltd, China). ProDH catalyzed dehydrogenation was detected by methyl isothiocyanate and measured at 600nm using a spectrophotometer.

### Proline Content Measurement

Proline was measured following established protocol (Bates et al., 1973) with minor modifications. Briefly, the fresh sample was weighed and cut into pieces. After adding 5ml 3% sulfur-salicylic acid in a 10ml centrifuge tube, it was boiled for 10min, and the supernatant was used for the proline extract. Two milliliter supernatant was reacted with 2ml glacial acid and 3ml acid ninhydrin, boiling for 45min. when the temperature of reaction mixture reached to room temperature, extracting with 5ml toluene. The supernatant was measured at 520nm, and proline content was calculated from a standard curve.

### RNA Isolation and Quantitative Real-Time RT-PCR

Total RNA was isolated from the leaves at different stages (i.e., elongation and reproductive stages) by the TRIzol reagent method (Invitrogen, Carlsbad, CA, USA) then subjected to reverse transcription with the PrimeScript RT reagent Kit (Takara, Shiga, Japan). SYBR Green (Takara, Shiga, Japan) was used as the reporter dye. Switchgrass ubiquitin1 (*PvUBQ1*) gene was used as the internal control. The relative expression levels of genes were calculated using the 2^−△△CT^ method (Livak and Schmittgen, 2001). Primers used for qRT-PCR are provided under **Table S1**.

### Salt Treatment

Salt treatment was performed according to (Jha et al., 2013). Briefly, the I3 leaves at R1 stage were cut into 4cm long pieces and soaked in either 0mM or 350mM NaCl solution for 30 days. Phenotypic changes of the leaf pieces were recorded. Furthermore, whole transgenic and WT plants with salinity stress in sand culture were also treated. Uniform tillers of both transgenic and control plants were treated with 1/2 × Hoagland nutrient solution supplemented with 0mM or 350mM NaCl, respectively, refreshed every two days. After 15 days, the researchers measured the physiological indexes (i.e., relative water content [RWC], electrolyte leakage [EL], chlorophyll, proline, Na^+^ and K^+^ contents, and root vigor). RWC and EL were measured following the methods (Li et al., 2010). Na^+^, K^+^ contents, and chlorophyll were measured following the methods as previously described (Li et al., 2010). Root vigor was evaluated following established protocol using the method of TTC (2, 3, 5-Tripheyl Tetrazolium Chloride) (Kausar et al., 2009). The roots were immersed and placed in the dark for 1 ~ 3h at 37°C, then they were ground with 3 to 4ml ethyl acetate and a small amount of quartz sand. Finally, the liquid was measured with spectrophotometer at wavelength of 485nm. Additionally, we also applied exogenous proline (10mM) to the RNAi transgenic plants to determine whether external proline could rescue their salt-sensitive phenotypes. SAMDC, SPDS and SPMS are the enzymes for PAs synthesis, and PAs are catabolized by PAO (Hanfrey et al., 2001; Shao et al., 2010). Based on the amino acid sequence of *AtSAMDC* (AT3G02470), *AtSPDS* (AT1G70310), *AtSPMS* (AT5G53120) and *AtPAO* (NM_105256.4) (Urano et al., 2003), the researchers BLAST-searched them in the Switchgrass Genome Annotation Program and found the full-length cDNA sequences of switchgrass *PvSAMDC*, *PvSPDS*, *PvSPMS* and *PvPAO*. Then we designed the primers used for qRT-PCR, and primers are provided under **Table S1**. Finally, we measured the relative expression level of *PvSAMDC* (Pavir.Ab02264.1), *PvSPDS* (Pavir.Ba02363.1), *PvSPMS* (Pavir.Ab01042.1) and *PvPAO* (Pavir.J39070.1) in the transgenic and WT plants.

### ROS Accumulation

H_2_O_2_ accumulation was evaluated by 3, 3ʹ-Diaminobenzidine (DAB) assay as described previously (Fryer et al., 2002). Additionally, H_2_O_2_ quantification was measured according to the method (Zhang et al., 2014). *PvCAT* and *PvSOD* play crucial role in ROS scavenging when plants experienced salt stress. Based on the amino acid sequence of *AtCAT* (AT1G20630) and *AtSOD* (AT1G08830) (Du et al., 2017), the researchers BLAST-searched them in the Switchgrass Genome Annotation Program and found the full-length cDNA sequences of switchgrass *PvCAT* (Pavir.J03636.1) and *PvSOD* (Pavir.Ib01670.1). Then we designed the primers used for qRT-PCR, and primers are provided under **Table S1**. Finally, we measured the relative expression level of *PvCAT* and *PvSOD* in the transgenic and WT plants.

### Statistical Analysis

Data from each trait were subjected to analysis of variance (ANOVA). The significance of treatments was tested at the level of *P* < 0.05. Standard errors were provided in all tables and figures when needed. All statistical analyses were performed using the SPSS statistical software package (SPSS 20.0, IBM Company, USA).

## Results

### Characterization of PvP5CS1 and PvP5CS2

**Figure 1:**
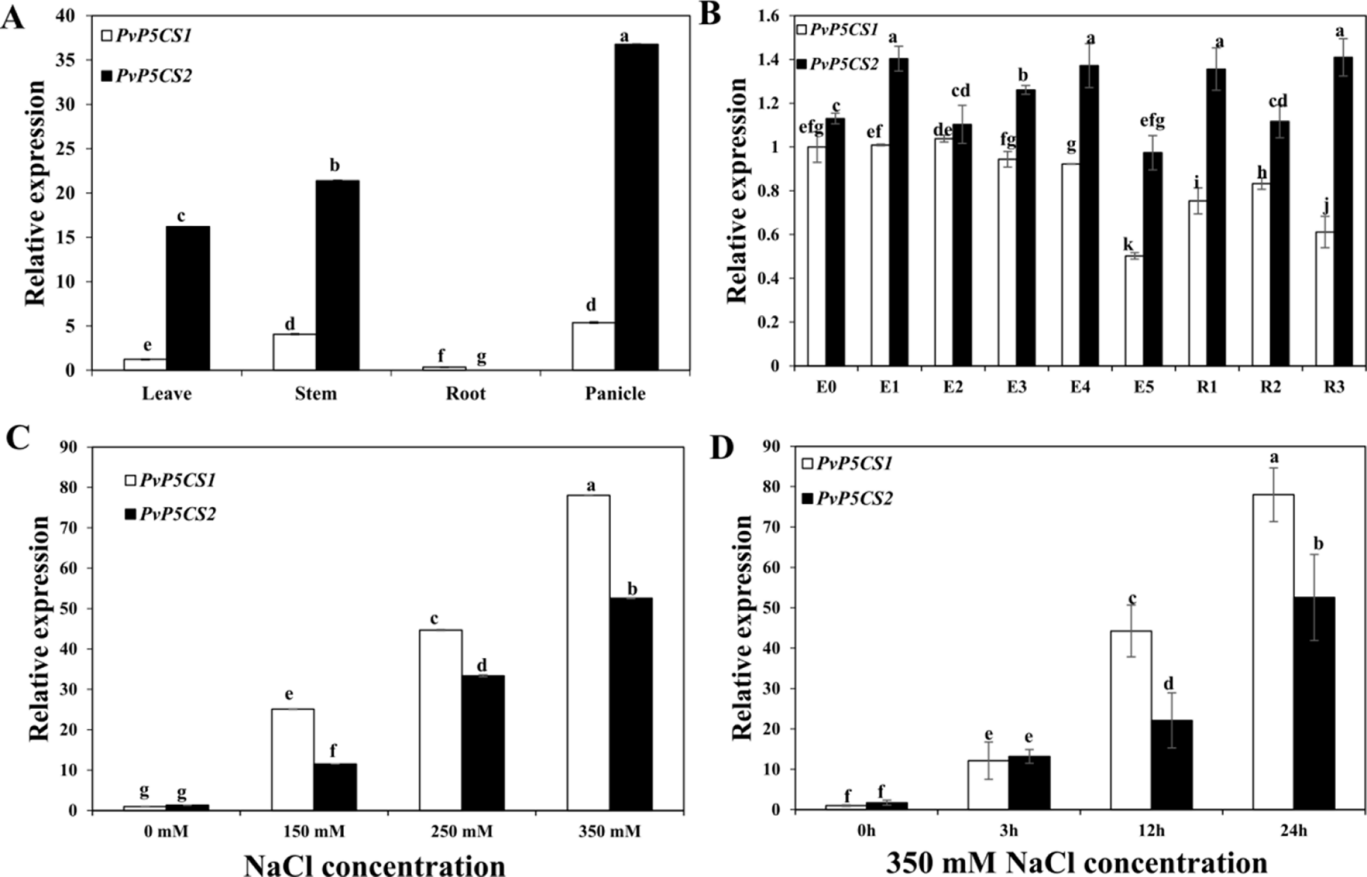
Expression pattern of two *PvP5CS* genes (*PvP5CS1* and *PvP5CS2*) in switchgrass. Relative expression level of two *PvP5CS* genes in different tissues **(A)**. Relative expression level of two *PvP5CS* genes in different development stages **(B)**. Analysis of *PvP5CS* genes expression under different NaCl concentrations (0 mM, 150mM, 250mM, and 350mM) after 24h salt treatment **(C)**. Transcripts of *PvP5CS* genes under 350 mM NaCl at the indicated time points **(D)**. Switchgrass *UBQ1* was used as the reference for normalization. At the first stage of elongation, there in none internode, so it is defined as E0. When switchgrass grows the internode, E1 to E5 are defined by the number of internodes present. At the beginning of the reproductive stage, flag leaf comes out, and it is defined as R1. When switchgrass has the fully emerged spikelets without peduncle, it arrived at R2 stage. The R3 stage is defined by fully emerged spikelets and visible peduncle. Data are mean values of three biological repeat, and significance of treatments was tested at the *P* < 0.05 level (one way ANOVA, Dunnett's test). WT: wild type control.

To understand how *PvP5CS*s function in switchgrass, we BLAST-searched the switchgrass genome referring to *Arabidopsis* sequences in the Phytozome version 12.1 and identified two putative *PvP5CS* candidate genes. Amino acid sequences of *P5CS* from NCBI were used to construct the phylogenetic tree. The cladogram was divided into three major groups (**Figure S1**). *P5CS* from monocots are clustered into two distinct groups (i.e., *P5CS1* and *P5CS2* groups), the result showed that *PvP5CS1* is clustered into P5CS1 and *PvP5CS2* is clustered into P5CS2. Next, the result of multiple sequence alignment showed that the sequence homology is 80.66% between *AtP5CS* and *PvP5CS* genes. Additionally, the conserved domains analysis showed that *PvP5CS2* has a specific hit that not existed in *PvP5CS1*, it is AAK_P5CS_ProBA (**Figure S1**), similar in *AtP5CS* genes. For further analyze the difference between the two *PvP5CS* genes, we described *PvP5CS1* and *PvP5CS2* using temporal and spatial expression patterns by reverse transcriptase quantitative PCR (RT-qPCR) (**Figure 1**). The results showed that *PvP5CS1* highly expressed in the stem and panicle while *PvP5CS2* highly expressed in the roots and panicle. Additionally, *PvP5CS1* was expressed higher at E0, E1, and E3 stages; while *PvP5CS2* showed higher expression at E4, R1 and R3 stages (**Figures 1A, B**). These results showed that the two *PvP5CS* genes have different expression patterns in the growth and development of switchgrass. To evaluate if the *P5CS* are related to salt tolerance, expression levels of two *PvP5CS* genes during different salt concentrations treatment were examined. The results showed that the expression level of both genes peaked at 350mM NaCl concentration. Besides, expression levels of *PvP5CS1* were higher than that of *PvP5CS2* at four different salt stress concentrations (**Figure 1C**). To further clarify the additional influence of salt stress on transcripts of *PvP5CSs*, the expression patterns of two *PvP5CS* genes at various time points under 350mM NaCl treatment were investigated and the results demonstrated that the expression levels of *PvP5CS1* and *PvP5CS2* were significantly induced by salt treatment (**Figure 1D**) thereby suggesting *PvP5CSs* response and function in salt treatment.

### Effect of PvP5CS1 and PvP5CS2 in Plant Growth and Development

**Figure 2:**
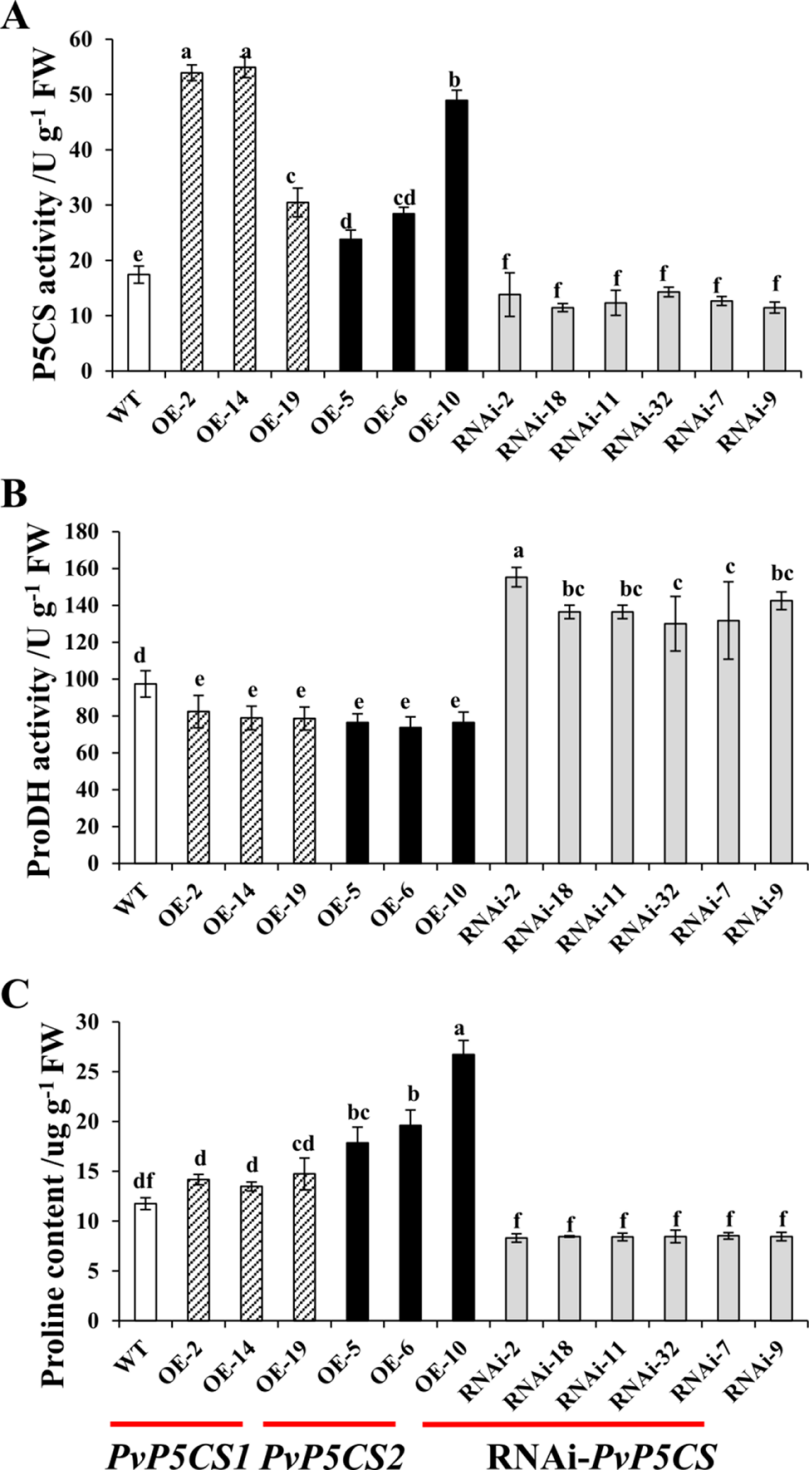
P5CS, ProDH activity and proline content in *PvP5CS OE* and *RNAi* transgenic plants. P5CS **(A)** and ProDH **(B)** activity and Proline content **(C)** in *PvP5CS OE* and *RNAi* transgenic plants. Data are mean values of three biological repeat, and significance of treatments was tested at the *P* < 0.05 level (one way ANOVA, Dunnett's test).

To evaluate the physiological functions and mechanisms of two *PvP5CS* genes, *PvP5CS1* and *PvP5CS2* overexpression lines and *PvP5CS*-RNAi lines were generated. A total of 26 transgenic lines (T0 generation) overexpressing *PvP5CS1*, 20 transgenic lines (T0 generation) overexpressing *PvP5CS2* and 39 *PvP5CS*-RNAi lines (T0 generation) were generated and tested as positive by genomic PCR analysis (**Figure S2**). In relation to WT plants, the expression levels of *PvP5CS1* and *PvP5CS2* were significantly higher in the most of *PvP5CS1* and *PvP5CS2* overexpression lines except OE-*PvP5CS1* line 6, and that were lower in the all *PvP5CS*-RNAi lines (**Figure S3**). According to their expression levels, the researchers selected three independent *PvP5CS1* overexpression lines (designated as *OE-2*, *OE-14,* and *OE-19*, respectively), three *PvP5CS2* overexpression lines (designated as *OE-5*, *OE-6,* and *OE-10*, respectively), and six independent RNAi lines (designated as *RNAi-2*, *RNAi-7*, *RNAi-9*, *RNAi-11*, *RNAi-18,* and *RNAi-32*, respectively) for further analysis. The selected OE and RNAi transgenic lines used for the experiment were labeled with the stars in the **Figure S3**. The enzyme activity of *P5CS* and proline content were higher in the *PvP5CS* overexpression lines but lower in *PvP5CS*-RNAi lines when compared to the WT plants (**Figures 2A, C**). The results showed that *PvP5CS* was highly expressed in the *PvP5CS* overexpression lines and downregulated in the *PvP5CS*-RNAi lines. Consequently, to further evaluate the effect of *P5CS* enzyme on proline metabolism, ProDH activity was tested in *PvP5CSs* overexpressing and in RNAi-*PvP5CS* lines. The result showed that the enzyme activity of ProDH was higher in the *PvP5CS*-RNAi lines and lower in the *PvP5CS1* and *PvP5CS2* overexpression lines. Thus, the proline metabolism is changed both in the overexpression lines and RNAi transgenic plants (**Figure 2B**).

**Figure 3:**
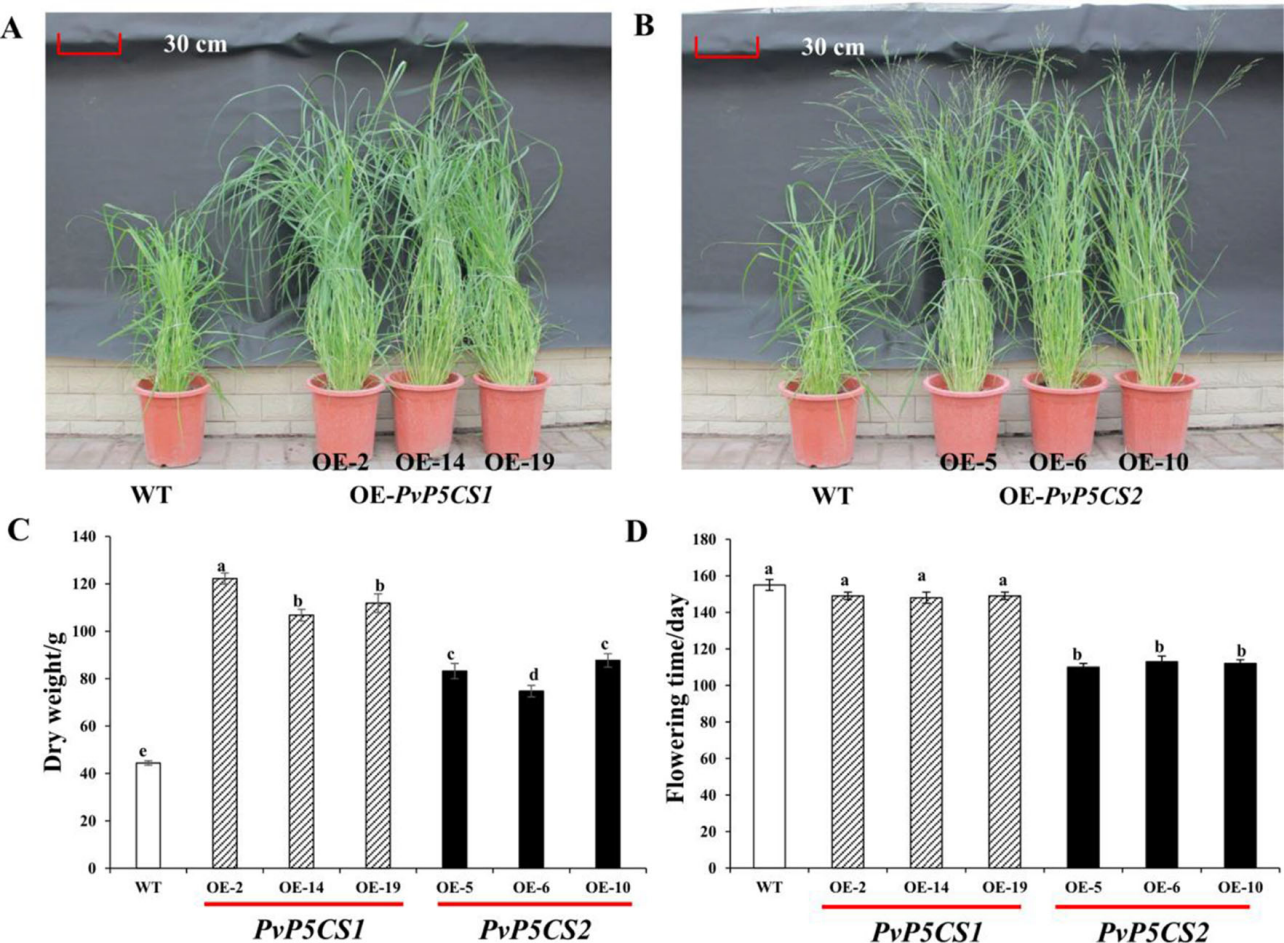
Phenotypic comparation and biomass analysis among *PvP5CS OE* transgenic plants. Phenotypes of three independent transgenic lines overexpressing *PvP5CS1* **(A)** and *PvP5CS2* **(B)**. Total above ground dry weight of transgenic lines overexpressing *PvP5CS* genes **(C)**. Flowering time in OE-*PvP5CS1* and OE-*PvP5CS2* transgenic switchgrass lines **(D)**. Data are mean values of three biological repeat, and significance of treatments was tested at the *P* < 0.05 level (one way ANOVA, Dunnett's test).

Interestingly, *PvP5CS* overexpression lines grew faster in greenhouses than WT plants. Phenotypic traits (i.e., plant height, tiller number, leaf length, internode length, and diameter) were measured in transgenic and WT plants (**Figures 3A, B**; **Table S2**). When compared to the WT, *PvP5CS1* and *PvP5CS2* overexpression lines showed on average 38.2% and 37.3% higher plant height, 30.3% and 21.5% more number of tillers, 51.1% and 34.9% longer leaf length, 58.1% and 47.4% wider internode diameter and 36.3% and 23.8% increase in internode length, respectively. The dry weight biomass of plants at five months old was measured and the result showed the biomass of *PvP5CS1* overexpression lines were 140–180% higher than WT plants while *PvP5CS2* overexpression lines were a 68–98% higher than WT (**Figure 3C**). Thus, both overexpressing *PvP5CS1* and *PvP5CS2* genes improve the biomass in transgenic switchgrass. In addition, it was found that the flowering time is different between transgenic and WT plants. The data showed that flowering time of *PvP5CS2* overexpression lines are shorter by an average of 30 days than WT plants while no obvious difference was manifested for the *PvP5CS1* overexpression lines (**Figure 3D**). To further evaluate the flowering related genes, we identified two flowering-related genes which are *PvFT* and *PvFLC* and the expression level of *PvFT* was higher in OE-*PvP5CS2* transgenic plants. However, the expression level of *PvFLC* was lower in both *PvP5CS1* and *PvP5CS2* overexpression lines (**Figure S4**). These results suggested that *PvP5CS2* gene may play an important role in regulating the flowering time in Switchgrass.

### PvP5CS1 and PvP5CS2 Positively Response to Salt Tolerance

**Figure 4:**
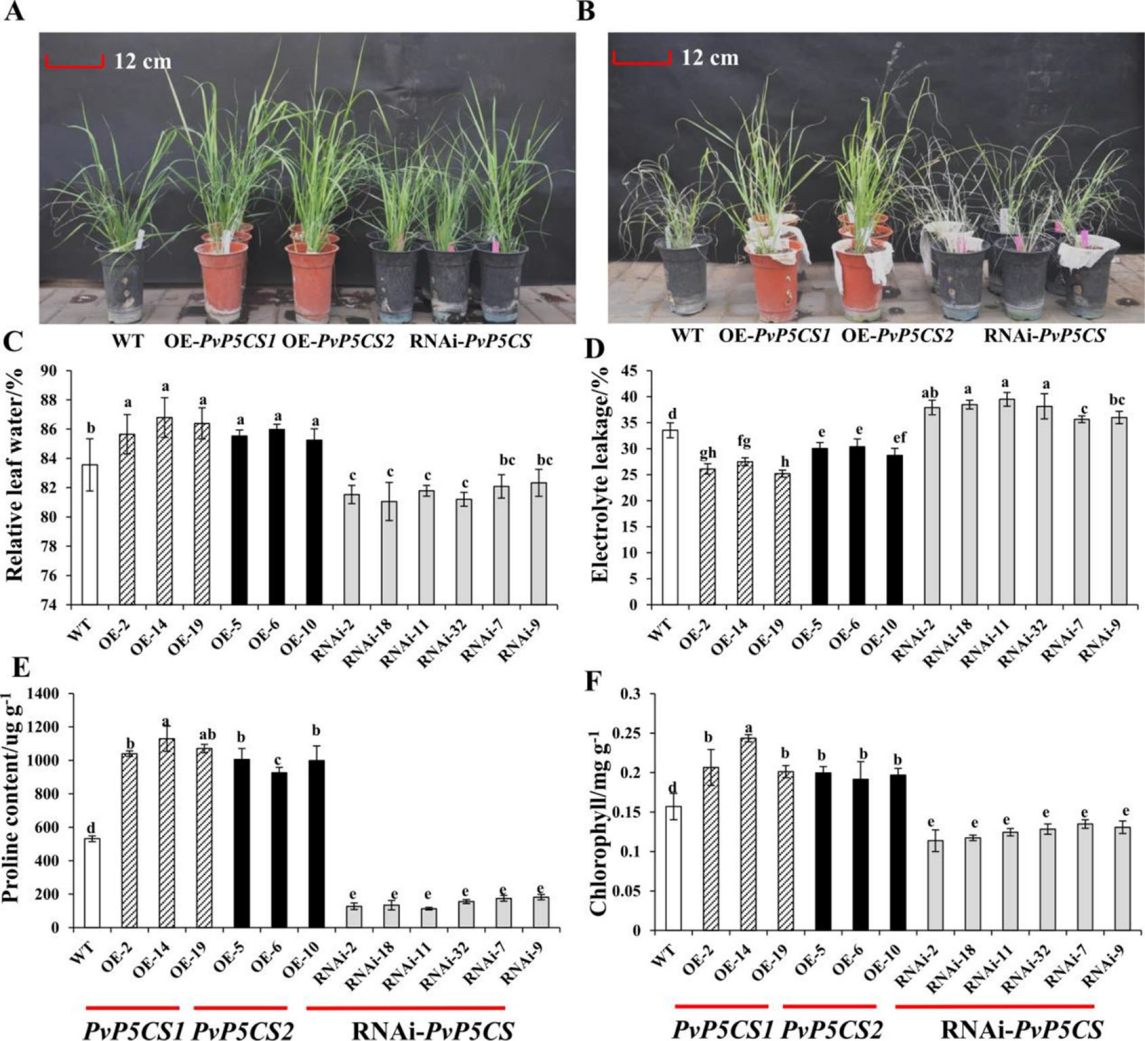
The salt tolerant evaluation of *PvP5CS OE* and *RNAi* transgenic plants under salt treatment. Phenotypes of *PvP5CS OE* and *RNAi* transgenic plants under 0 mM NaCl **(A)** and 350 mM NaCl **(B)** treatment for 15 days. Relative leaf water content **(C)**, Electrolyte leakage **(D)**, proline **(E)** and Chlorophyll **(F)** of transgenic lines OE-*PvP5CS1*, OE-*PvP5CS2* and RNAi-*PvP5CS* in **(B)** after 350 mM NaCl treatment for 15 days. Data are mean values of three biological repeat, and significance of treatments was tested at the *P* < 0.05 level (one way ANOVA, Dunnett's test).

Based on the previous results that the expression levels of *PvP5CSs* were upregulated under salt stress, it was theorized that the two *PvP5CS* genes may play the significant role in salt stress response. To verify this hypothesis, salt tolerance experiments were performed on *PvP5CS* overexpression and RNAi transgenic switchgrass. After 30 days, leaves of the WT plants almost turned yellow with the overexpression lines losing their green color under 350mM NaCl salt stress (**Figure S5**). Taken together, *PvP5CS1* and *PvP5CS2* overexpression lines are involved in salt tolerance. Additionally, this result is supported by less chlorophyll bleaching in the cut-leaf float assay while large areas of leaves of RNAi transgenic lines lose their green color. However, there are no obvious phenotypic difference between the WT, *PvP5CS* overexpression and RNAi transgenic plants under normal growth conditions (**Figure 4A**). Only when treated with 350mM NaCl, the WT and *PvP5CS*-RNAi transgenic plants showed serious leaf damage after 15 days whereas *PvP5CS* OE-transgenic lines retained its green color and showed better performance (**Figure 4B**). This suggested that the PvP5CS1 and PvP5CS2 genes are involved in salt tolerance.

To see the effect of salt stress on plasma membrane integrity and membrane permeability, we measured the relative water content and electrolyte leakage. The result showed, there was no difference in RWC and EL in all lines under normal condition (**Figures S6A, B**). However, under salt-stressed condition, *PvP5CS* overexpressing lines were shown to contain less RWC and EL than the WT and *PvP5CS*-RNAi transgenic plants (**Figures 4C, D**). Meanwhile, Chlorophyll content was indistinguishable between WT and *PvP5CS1* overexpression transgenic plants under normal condition while RNAi transgenic plants had relatively lower chlorophyll content (**Figure S6C**). When treated by salt stress, chlorophyll content decreased sharply in RNAi transgenic plants compared to WT plants (**Figure 4F**). Additionally, it was found that ion imbalance, especially intracellular K^+^ and Na^+^, directly affect carbon fixation and biomass production in plants under salt stress. Shoots Na^+^ and K^+^ contents in transgenic and WT plants were measured. Na^+^ and K^+^ contents in shoots were similar between transgenic and WT plants under normal conditions. When treated with 350mM salt stress, RNAi transgenic lines accumulated more Na^+^ but lower K^+^ content. Na^+^ content was lower and K^+^ content was higher in the *PvP5CS* overexpression transgenic lines relative to WT plants (**Figure S7**). Enrichment of proline significantly altered salt stress response which also happened in *PvP5CS* overexpression lines. Proline content in *PvP5CS* overexpression lines showed approximately a 2-fold increase comparing to WT, but around 4-fold decrease in RNAi transformants (**Figure 4E**). These results suggest that upregulated expression of *PvP5CS1* and *PvP5CS2* is critical for salt stress tolerance.

### PvP5CS OE-Transgenic Plants Sustained Root Vigor Through Regulating Na^+^ and K^+^ Contents Under Salt Stress

**Figure 5:**
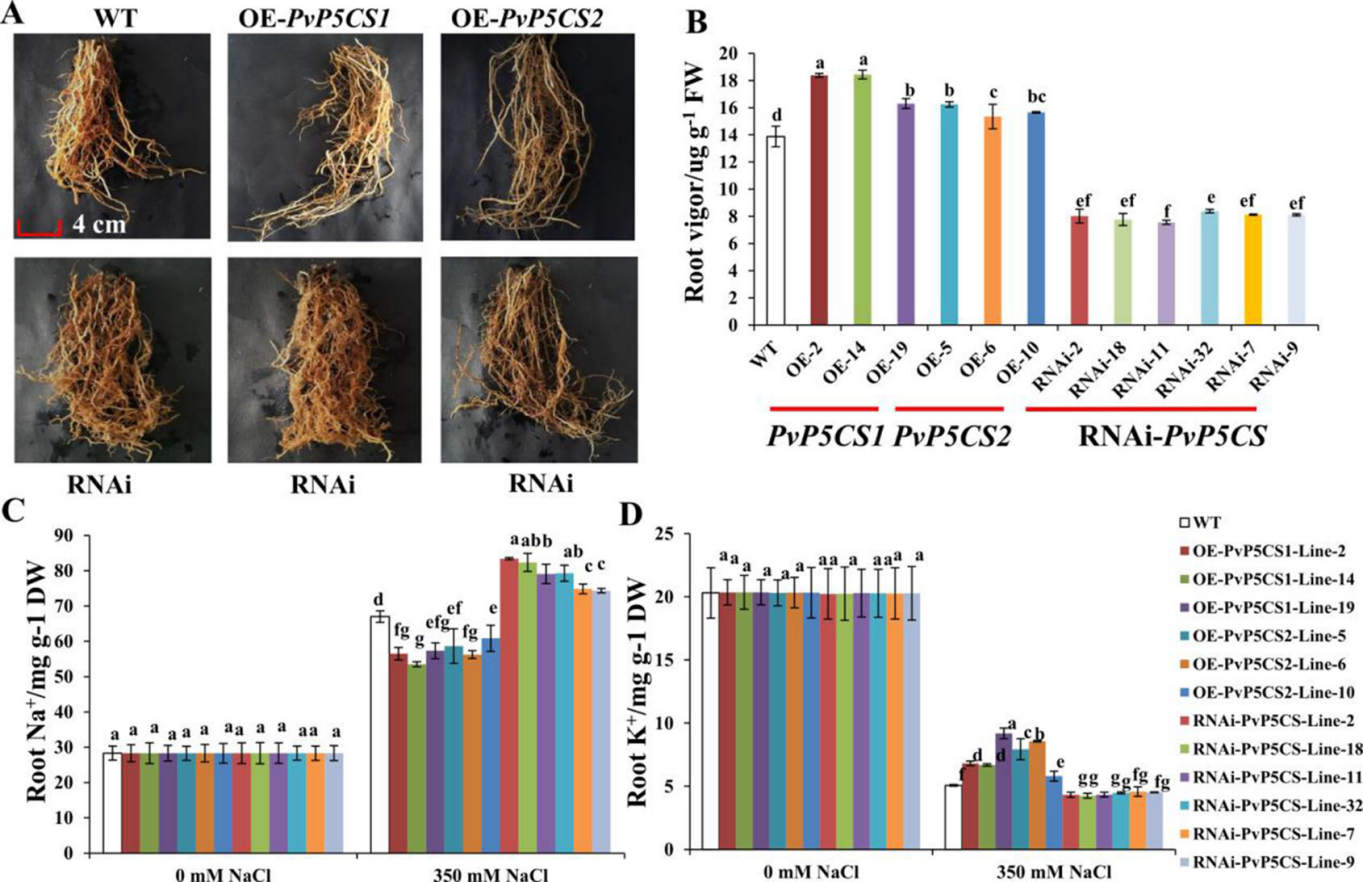
Phenotype and ion (Na^+^ and K^+^) contents of roots in *PvP5CS OE* and *RNAi* transgenic plants under salt stress. Phenotype of roots in *PvP5CS OE* and *RNAi* transgenic plants under 350 mM NaCl salt stress **(A)**. Root vigor in *PvP5CS OE* and *RNAi* transgenic plants under 350 mM NaCl salt stress **(B)**. Na^+^ **(C)** and K^+^ **(D)** contents of roots in *PvP5CS OE* and *RNAi* transgenic plants under 350 mM NaCl salt stress. Data are mean values of three biological repeat, and significance of treatments was tested at the *P* < 0.05 level (one way ANOVA, Dunnett's test).

Salt stress leads to serious damage to roots thereby affecting the growth and arrangement of roots (Julkowska and Testerink, 2015). To evaluate whether the *PvP5CS* genes have relationship with root development during salt stress, the growth situation of transgenic plants was tested. The roots of *PvP5CS1* and *PvP5CS2* overexpression transgenic lines survived under salt stress. In contrast, the growth of roots in RNAi transgenic lines were suppressed and a different arrangement was formed (**Figure 5A**). To further analyze the extent of root damage under salt stress, the root vigor was measured. Root vigor of overexpression transgenic lines was higher than the ones in WT plants. An approximate increase of 21% in *PvP5CS1* overexpression transgenic lines and 17% in *PvP5CS2* overexpression transgenic lines were observed, while the *PvP5CS*-*RNAi* transgenic plants showed an approximate 43% decrease compared to WT plants (**Figure 5B**). Moreover, to see the relationship between salt stress with Na^+^ and K^+^, we measured the Na^+^ and K^+^ contents in roots and the result revealed no difference in Na^+^ and K^+^ contents in roots in normal condition. However, in those under the salt-stressed conditions, Na^+^ accumulation in roots increased significantly in all transgenic plants and WT while the K^+^ levels in roots started to decline (**Figures 5C, D**). In relation to the wild type, the increase in Na^+^ content was lower by more than 32% and 23% in the roots of *PvP5CS1* and *PvP5CS2* overexpression transgenic plants, respectively. Conversely, the increase in Na^+^ content was higher by more than 28% in the roots of RNAi transgenic lines (**Figure 5C**). Similarly, the decrease in the K^+^ content was faster by more than 7% in the roots of RNAi transgenic lines while the decrease in the K^+^ content was slower by 13% and 14% in the roots of *PvP5CS1* and *PvP5CS2* transgenic plants, respectively, compared to WT plants (**Figure 5D**). These results indicated that there is a significant change of Na^+^ and K^+^ contents between transgenic and WT plants. These further suggest that *PvP5CS1* and *PvP5CS2* overexpression transgenic lines reduced root damage by regulating Na^+^ and K^+^ contents under salt stress.

### PvP5CS OE-Transgenic Lines Accumulate Less ROS and the RNAi Lines Accumulate More ROS Under Salt Stress

**Figure 6:**
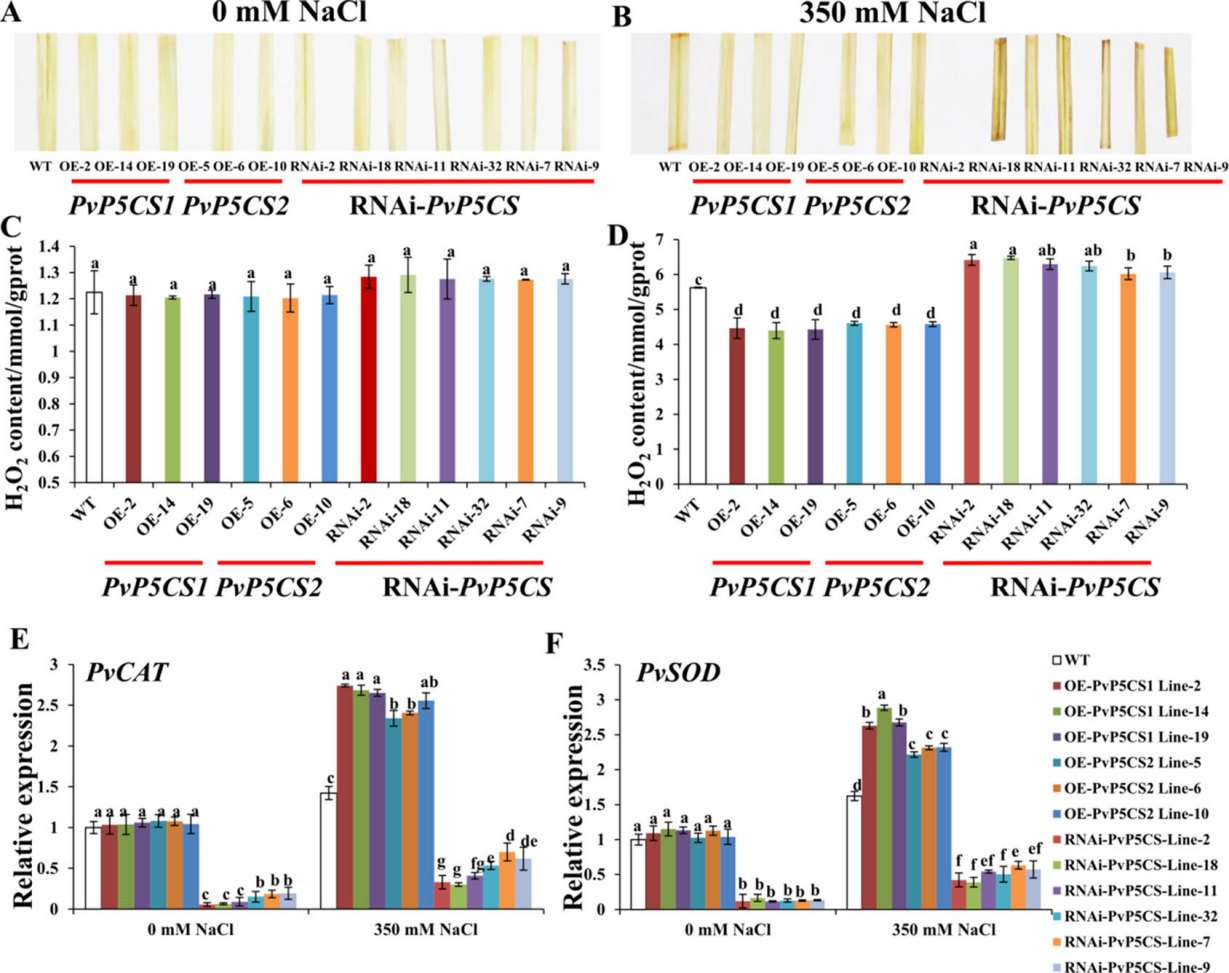
Overexpression of *PvP5CS* genes in switchgrass positively regulates the induction of ROS scavengers and membrane transporters, and reduces H_2_O_2_ accumulation under 350 mM NaCl treatment. Color of detached leaves after staining with diaminobenzidine (DAB) in wild type, *PvP5CS OE* and *RNAi* transgenic plants under 0 mM **(A)** and 350 mM **(B)** NaCl treatment. H_2_O_2_ content in the leaves of wild type, *PvP5CS OE* and *RNAi* transgenic plants under 0 mM NaCl **(C)** and 350 mM NaCl **(D)** treatment. Relative expression levels of *PvCAT* **(E)** and *PvSOD* **(F)** in wild type, *PvP5CS OE* and *RNAi* transgenic plants under 0 and 350 mM NaCl treatment. Data are mean values of three biological repeat, and significance of treatments was tested at the *P* < 0.05 level (one way ANOVA, Dunnett's test).

ROS is one of the most important indicators of salt response. It can be analyzed by histochemical staining with DAB. Histochemical results did not show significant brown precipitations in all transgenic and WT plants under normal conditions (**Figure 6A**). Conversely, under salt-stressed conditions, the intensity of brown precipitations in WT leaves was stronger than those in *PvP5CS* OE-transgenic plants, but weaker than in RNAi transgenic plants (**Figure 6B**). To verify the histochemical staining, the levels of H_2_O_2_ were quantified using a detection kit. In consonance with the histochemical staining results, quantitative measurement showed that H_2_O_2_ levels were similar between the transgenic and WT plants under normal conditions (**Figure 6C**). While the levels of H_2_O_2_ in OE-transgenic lines were significantly lower than those in WT plants during the salt treatment, the H_2_O_2_ content in RNAi transgenic plants were significantly higher than in WT plants (**Figure 6D**). These results demonstrate the function of *PvP5CSs* in salt tolerance through ROS pathway. To confirm whether accumulation of ROS causes plant self-defensive ability, the transcript levels of two important enzyme genes (i.e., *PvCAT* and *PvSOD*) which play crucial role in ROS scavenging when plants experienced salt stress were examined. The transcription levels of *PvCAT* and *PvSOD* in *PvP5CS* OE-transgenic lines had no significant difference with WT under normal conditions. Meanwhile, the expression levels of *PvCAT* and *PvSOD* were significantly decreased in *PvP5CS*-RNAi transgenic plants compared to those in WT. Under salt stress, expression levels of *PvCAT* and *PvSOD* were significantly upregulated in *PvP5CS* OE-transgenic lines, but downregulated in *PvP5CS*-RNAi transgenic plants compared to WT plants (**Figures 6E, F**).

### Alteration of Transcript Levels of Spd and Spm Synthesis and Metabolism Genes in PvP5CS Overexpression and RNAi Transgenic Lines

**Figure 7:**
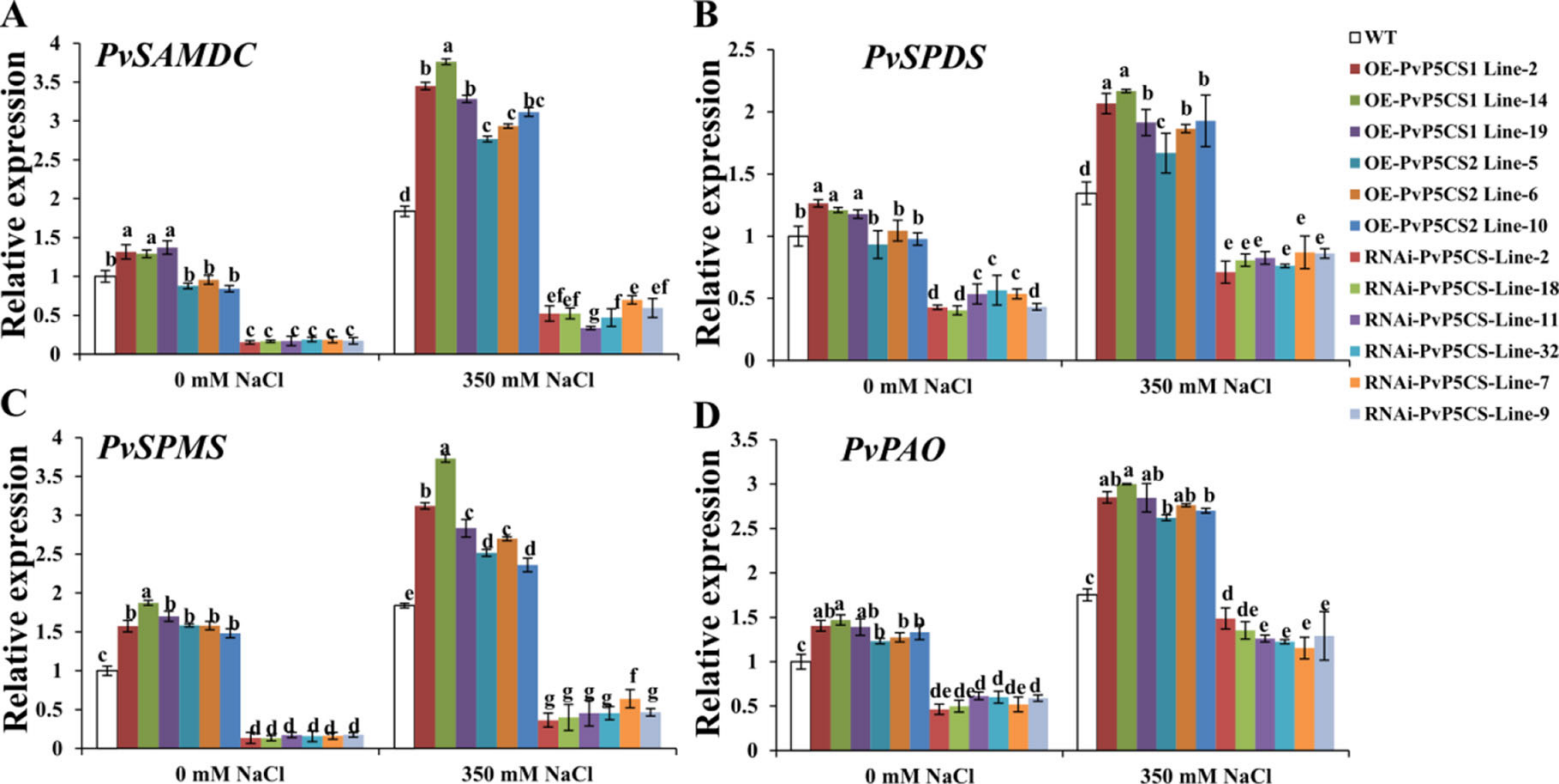
Transcript levels of the genes involved in polyamine synthesis **(A–C)** and catabolism **(D)** in *PvP5CS OE* and *RNAi* transgenic plants under control and salt stress conditions. Switchgrass *UBQ1* was used as the reference for normalization. Data are mean values of three biological repeat, and significance of treatments was tested at the *P* < 0.05 level (one way ANOVA, Dunnett's test).

The researchers then further elucidated the underlying physiological mechanism relative to enhanced salt tolerance in the *PvP5CS* OE-transgenic plants. Polyamine is involved in adaptation to environmental signals (Alcázar et al., 2010). SAMDC, SPDS and SPMS are the enzymes for PAs synthesis, and PAs are catabolized by PAO (Hanfrey et al., 2001; Shao et al., 2010).The expression levels of these genes were examined by qRT-PCR. *PvSAMDC*, *PvSPDS*, *PvSPMS*, and *PvPAO* were all upregulated in *PvP5CS1* overexpression transgenic lines under normal conditions, but only *PvSPMS* and *PvPAO* genes were elevated in the *PvP5CS2* overexpression transgenic lines with all other genes decreasing in *PvP5CS*-RNAi transgenic plants compared to WT plants (**Figure 7**). Furthermore, Spd and Spm synthesis and metabolism related genes were induced by salt treatment in all plants, but the elevation in *PvP5CS OE* transgenic lines was much higher than that in WT plants while the opposite happened in *PvP5CS*-RNAi transgenic plants (**Figure 7**).

**Figure 8:**
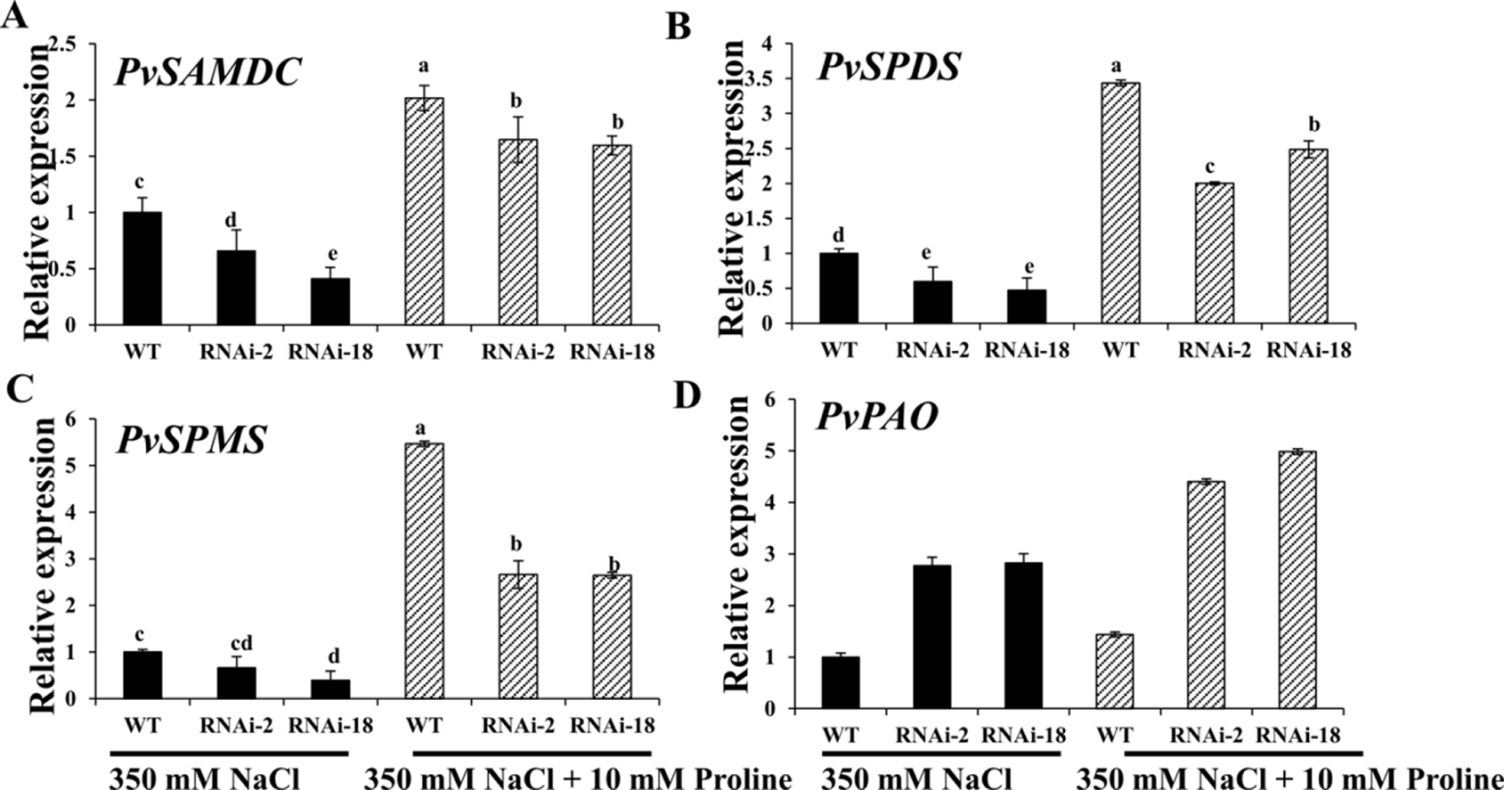
Exogenous proline can largely improve transcript levels of the genes involved in polyamine synthesis **(A–C)** and catabolism **(D)** in *PvP5CS RNAi* transgenic plants under 350 mM NaCl salt stress. Switchgrass *UBQ1* was used as the reference for normalization. Data are mean values of three biological repeat, and significance of treatments was tested at the *P* < 0.05 level (one way ANOVA, Dunnett's test).

The researchers then attempted to analyze whether expression of polyamine synthesis genes was altered in *PvP5CS*-RNAi transgenic plants under 350mM salt stress with exogenous application of proline. Compared with the control (i.e., no exogenous proline under salt stress), transcript levels of Spd and Spm synthesis and metabolism genes in both *PvP5CS*-RNAi transgenic and WT plants were significantly increased under salt stress when applied with 10mM proline, but the extent of increase in *PvP5CS*-RNAi transgenic plants were much lower than that in WT plants (**Figure 8**). These results showed that exogenous proline could accelerate the expression levels of polyamines metabolism-related genes under salt stress. In summary, these results highlight the function of *PvP5CS* in Spd and Spm synthesis and metabolism during salt response.

## Discussion

### Function of PvP5CS1 and PvP5CS2 in Switchgrass

P5CS is a rate limiting enzyme in proline biosynthesis (Székely et al., 2008). Generally, P5CS enzyme is encoded by two homologous *P5CS* genes. *P5CS* genes have been isolated from various plants species including one housekeeping and one stress-specific P5CS isoform. Although the duplicated *P5CS* genes share a high level sequence homology in coding regions, their transcriptional regulations are different (Szabados and Savoure, 2010). In *Arabidopsis*, *P5CS1-GFP* is normally localized in cytosol of leaf mesophyll cells and *P5CS2-GFP* is predominantly localized in the cytosol. When cells are exposed to salt or osmotic stress, proline biosynthesis is regulated by the *P5CS1* enzyme and its transcript is induced by different abiotic stress in the chloroplasts (Székely et al., 2008). Moreover, in rice although both *OsP5CSs* genes are upregulated by exogenous ABA, their kinetics differ with a faster and stronger upregulation of *OsP5CS1* transcripts compared to that of *OsP5CS2* in seedlings and different tissues of rice plants (Hur et al., 2004; Sripinyowanich et al., 2013). In switchgrass, *PvP5CS1* and *PvP5CS2* were induced by salt stress, whereas the transcript levels of *PvP5CS1* showed a faster and stronger upregulation compared to the transcript levels of *PvP5CS2*. Furthermore, *PvP5CS1* and *PvP5CS2*, were found to differ in tissue tropism and the kinetics and levels of transcript expression, where the *PvP5CS1* showed higher expression at E0, E1, and E3 stages; while *PvP5CS2* showed higher expression at R1 and R3 (**Figures 1A, B**). The expression patterns indicate that two switchgrass *P5CS* paralogs might have different functions.

Under normal conditions, change in free proline content was accompanied by plant growth and development especially in reproductive organs (Lehmann et al., 2010). To explore the extent of functional diversification of duplicated *PvP5CSs* genes in switchgrass, we evaluated the genetic and physiological characterizations of *PvP5CS1* and *PvP5CS2* overexpression transgenic lines for different developmental stages of the plant. *PvP5CS2 OE* lines bloomed earlier, and this result is coupled with a significant difference in relative expression of flowering-related genes *PvFLC* and *PvFT* between *PvP5CS2 OE* and WT plants. On the other hand, the promoter of *Arabidopsis* P5CS2 was an early target of CONSTANS (CO), which is involved in floral transition (Samach et al., 2000). And later, concurrent silencing of two *AtP5CS* genes resulted in stunted growth and resulted late flowering (Nanjo et al., 1999). Consequently, *atp5cs2* mutant plants displayed a stronger late-flowering phenotype compared to that of *atp5cs1* mutant plants, and only the embryos in *atp5cs2 mutant plants* showed alterations of the cellular division planes and inactive growth (Mattioli et al., 2009). Furthermore, under long-day conditions, *atp5cs2* mutant plants were retarded in vegetative development whereas the growth and development of *atp5cs1* mutant plants was normal (Funck et al., 2012). Together with these findings, our results suggested that *PvP5CS2* also functions in reproductive growth stage in switchgrass. Nonetheless, it was also found that both *PvP5CS1* and *PvP5CS2 OE* plants improved salt stress under high salt treatment. These results indicate that proline generates the suppression of NaCl-induced physiological damage as shown by the lower electrolyte leakage and ROS levels which further explain that proline significantly improves the physiological status of stressed plants (**Figures 4** and **6**). Similarly, our previous results also showed that overexpressing heterologous P5CS genes from *Lolium perenne* and *Puccinellia chinampoensis* in switchgrass improve the salt tolerance by reducing the electrolyte leakage and ROS levels (Guan et al., 2018; Guan et al., 2019). Therefore, no matter overexpressing heterologous or endogenous P5CS genes in switchgrass, which can increase the proline content and improve the salt stress. Additionally, increased proline content also improved the plant growth and development, especially in flowering time and biomass.

### Proline Synthesis Cooperated With Polyamine Synthesis and Metabolism to Improve Salt Tolerance in Transgenic Plants

Reactive oxygen species is highly accumulated under stress conditions and endogenous plant cell sensors by recognizing the composite signaling system and transfers to kinases by secondary messengers (Waszczak et al., 2018). And also, the upregulation of proline biosynthesis results metabolic redistribution (Majumdar et al., 2016). Additionally, Polyamines also play essential roles in plant stress tolerance which partially reversed the NaCl-induced phenotypic and reprogramed the oxidative status (Tanou et al., 2014). Ornithine is a common precursor of PAs and proline and in plants, and putrescine can be synthesized from ornithine by ornithine decarboxylase (ODC). Despite lower concentrations of ornithine compared to PAs, Pro, and Glu, ornithine remains a gatekeeper in controlling PAs, Pro, and GABA Biosynthesis (Majumdar et al., 2016). Moreover, alteration of ornithine into PA biosynthesis affects the proline biosynthesis. Thus, it is meaningful to consider the relationship between PAs and proline metabolisms. Overexpressing *MfERF1* induced proline and polyamine enrichment under cold tolerance, suggesting that proline and PAs jointly respond and improve cold tolerance (Zhuo et al., 2018). The results showed that overexpression of *PvP5CS1* and *PvP5CS2* elevate the transcript levels of Spd and Spm synthesis and metabolism genes under normal and salt stress conditions (**Figure 7**). Additionally, *atpao5-2* and *atpao5-3* mutants (mutants of Spd and Spm metabolism gene PAO) were found to exhibit higher proline biosynthesis and salt tolerance, and the proline content is higher in mutants under salt stress (Zarza et al., 2017). Thus, increased proline biosynthesis is directly sourced from glutamate by *P5CS* and the pathway from ornithine to proline is suppressed which divert more ornithine into PAs biosynthesis. On the other hand, to illuminate the mechanical connection between external proline application and PA metabolism under salt stress, we measured the expression levels of PAs biosynthesis and catabolism genes in leaves. By comparing proline plus NaCl treatment with NaCl-only treatment, application of proline leads to enhanced transcript levels of PAs metabolism related genes both in *PvP5CS RNAi* and in WT plants compared to NaCl-alone treatment, and the improved degree in *PvP5CS RNAi* plants is higher than in WT plants (**Figure 8**) indicating that PA metabolism is regulated by external proline application.

**Figure 9:**
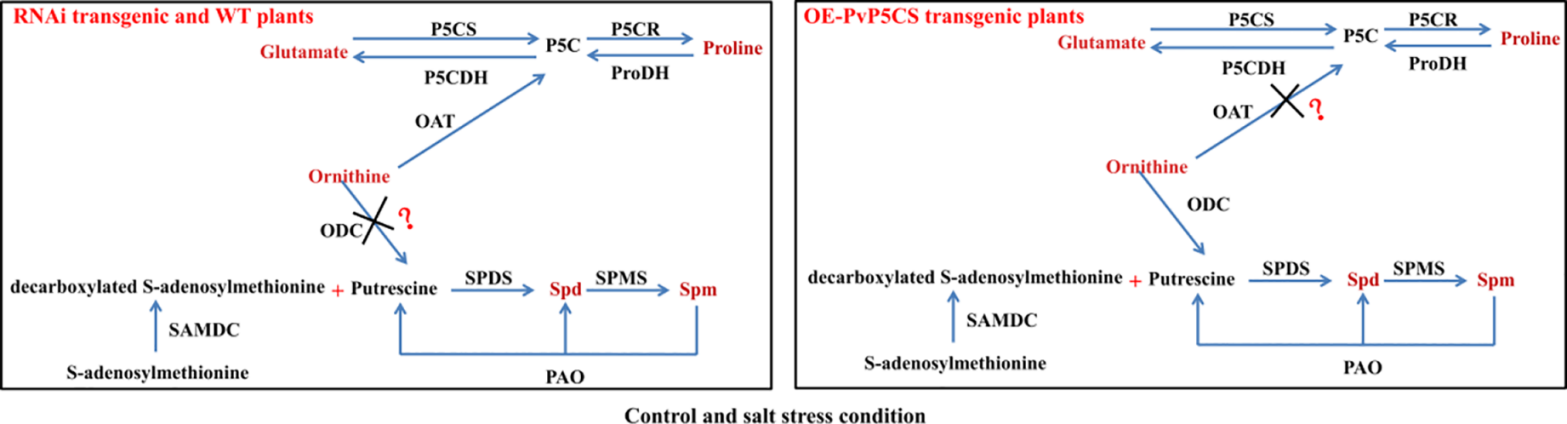
Schematic illustration of a working model depicting the relationship between proline and polyamine in wild type, *PvP5CS OE* and *RNAi* transgenic plants under control and salt stress conditions. In summary, proline metabolism is in cooperation with polyamines metabolism in switchgrass. If switchgrass has enough proline to confer salt stress, ornithine could be as substrate involved in PAs biosynthesis. However, under less proline accumulation to improve the salt tolerance of switchgrass, ornithine will be used as a precursor entering the pathway of proline synthesis. Altogether, proline and polyamine metabolism cooperated to improve salt tolerance in switchgrass.

Based on the results, the researchers were able to summarize the model of the connections between PAs and proline metabolisms under normal and salt stress (**Figure 9**). Ornithine is a key metabolite involving in proline and PAs metabolism. In the ornithine pathway, ornithine is transmitted by ornithine-delta-aminotransferase (OAT) producing GSA and P5C which is later converted to proline (Roosens et al., 1998). PAs biosynthesis is controlled by enzymes activity and substrate. Ornithine is the substrate of Put biosynthesis and transmitted by ornithine decarboxylase (ODC) producing Put, then Put is converted to Spd and Spm. In our results, under both normal and stressed conditions, ornithine as a precursor may enter pathway of proline synthesis, and proline metabolism plays the main part compared with PAs metabolism in *PvP5CS RNAi* and WT plants. Overexpression of *PvP5CS1* and *PvP5CS2* improved the proline biosynthesis under normal and salt stress conditions, and an excess of proline produces toxic action to plants which result in ornithine, as a precursor, entering the synthesis pathway of PAs. In summary, proline metabolism is in cooperation with polyamines metabolism in switchgrass. If switchgrass has enough proline to confer salt stress, ornithine could be as substrate involved in PAs biosynthesis. However, under less proline accumulation to improve the salt tolerance of switchgrass, ornithine will be used as a precursor entering the pathway of proline synthesis. Altogether, proline and polyamine metabolism cooperated to improve salt tolerance in switchgrass.

## Data Availability Statement

Publicly available datasets were analyzed in this study. The Gene sequences are downloaded from Switchgrass Genome Annotation Program.

## Author Contributions

Conceived and designed experiment: Y-WZ. Performed the experiments: CG, XC, H-YL, XL, and M-QL. Analyzed the data: CG, XC, H-YL, XL, M-QL, and Y-WZ. Wrote the manuscript: CG and YW-Z. All authors reviewed and approved the final manuscript.

## Conflict of Interest

The authors declare that the research was conducted in the absence of any commercial or financial relationships that could be construed as a potential conflict of interest.
