# Supplementary material for: Proline Biosynthesis Enzyme Genes Confer Salt Tolerance to Switchgrass (Panicum virgatum L.) in Cooperation With Polyamines Metabolism
Source: Front Plant Sci. 2020 Feb 14;11:46. doi: 10.3389/fpls.2020.00046 (PMC7033549; doi:10.3389/fpls.2020.00046)
Supplement: Supplementary file 1 [file DataSheet_1.docx]

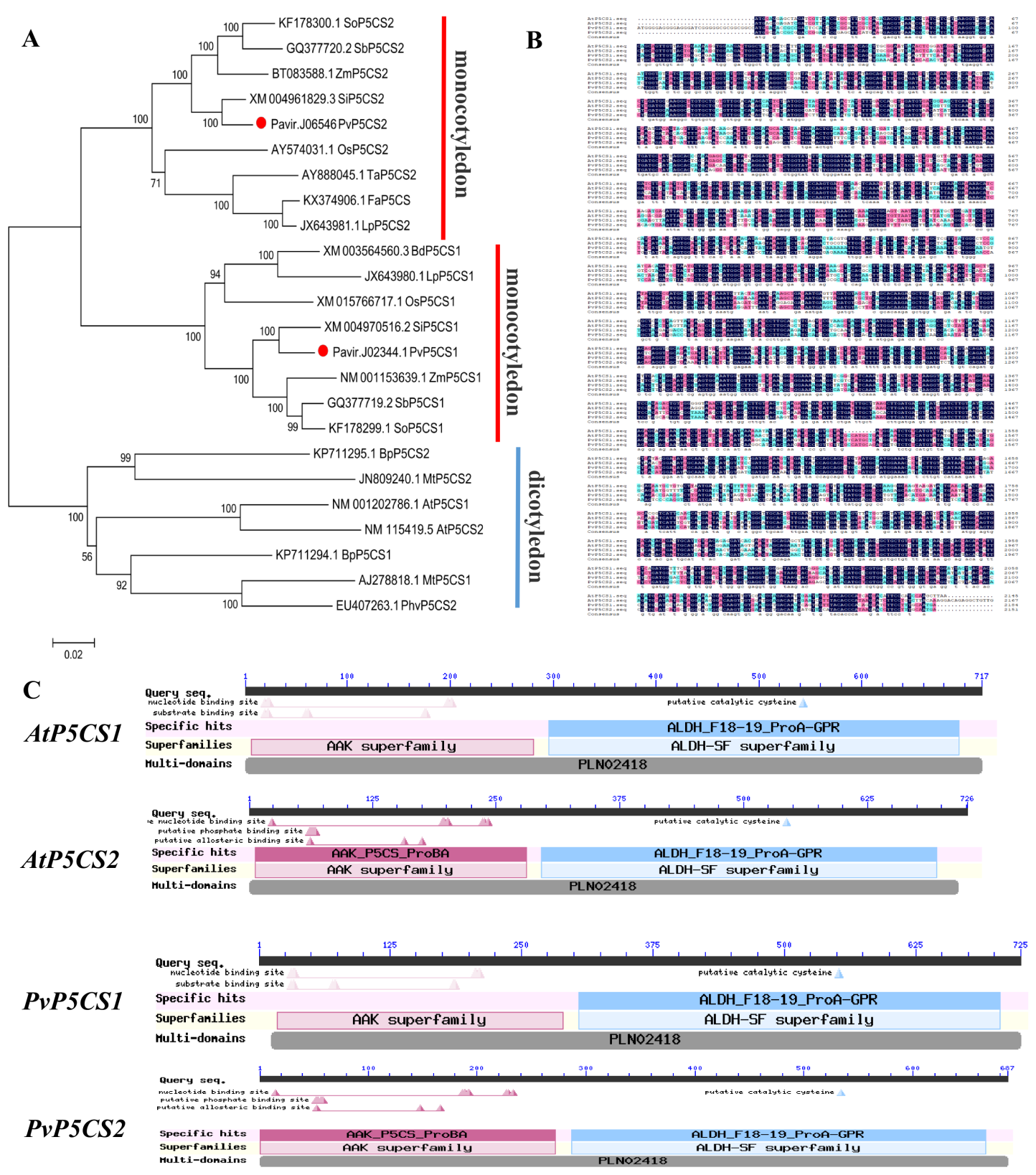


Figure S1. Bioinformation analysis. Phylogenetic analysis (A). cDNA sequence alignment of *AtP5CS1*, *AtP5CS2*, *PvP5CS1* and *PvP5CS2* (B). Conserved domain structure of protein (C).

SoP5CS2, *Saccharum*, KF178300.1; SbP5CS2, *Sorghum bicolor,* GQ377720.2; ZmP5CS2, *Zea mays*, BT083588.1; SiP5CS2, *Setaria italica*, XM_004961829; PvP5CS2, *Panicum virgatum*, Pavir.J06564; OsP5CS2, *Oryza sativa*, AY574031.1; TaP5CS2, *Triticum aestivum*, AY888045.1; FaP5CS, *Festuca arundinacea*, KX374906.1; LpP5CS2, *Lolium perenne*, JX643981.1; BdP5CS1, *Brachypodium distachyon*, XM_003564560.3; LpP5CS1, *Lolium perenne*, JX643980.1; OsP5CS1, *Oryza sativa*, XM_015766717.1; SiP5CS1, *Setaria italic*, XM_004970516.2; PvP5CS1, Pavir.J02344.1; ZmP5CS1, *Zea mays*, NM_001153639.1; SbP5CS1, *Sorghum bicolor,* GQ377719.2; SoP5CS1, *Saccharum*, KF178299.1; BpP5CS2, *Betula platyphylla*, KP711295.1; MtP5CS2, *Medicago truncatula*, JN809240.1; AtP5CS1, *Arabidopsis thaliana*, NM_001202786.1; AtP5CS2, *Arabidopsis thaliana*, NM_115419.5; BpP5CS1, *Betula platyphylla*, KP711294.1; MtP5CS1, *Medicago truncatula*, AJ278818.1; PhvP5CS2, *Phaseolus vulgaris*, EU407263.1.


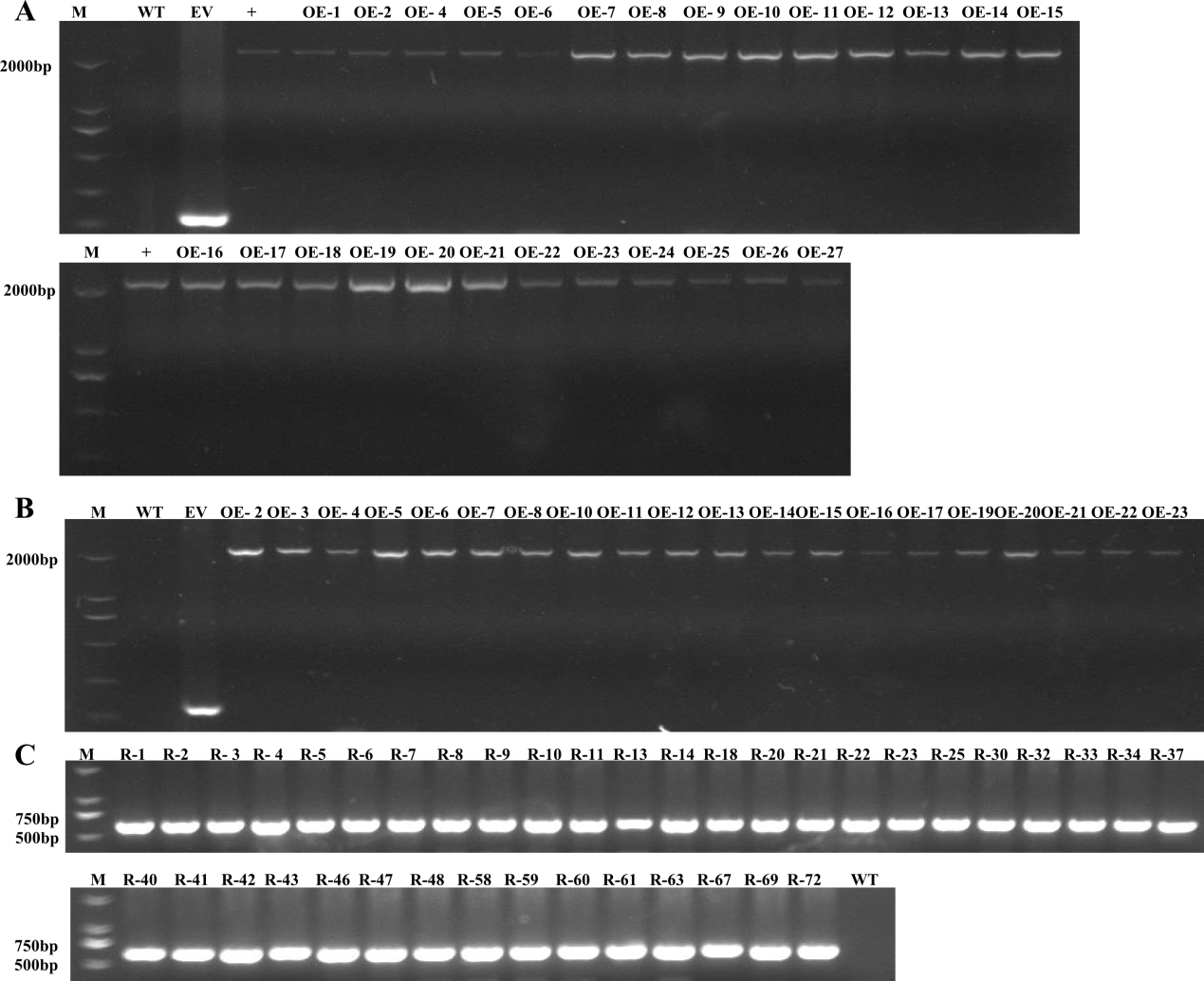


Figure S2. PCR identification. Transgenic plant overexpressing *PvP5CS1* genes (A). Transgenic plant overexpressing *PvP5CS2* genes (B). RNAi-*PvP5CS* transgenic plants (C). R-1 to R-72 represents the number of RNAi transgenic plants. M：DL2000 marker.


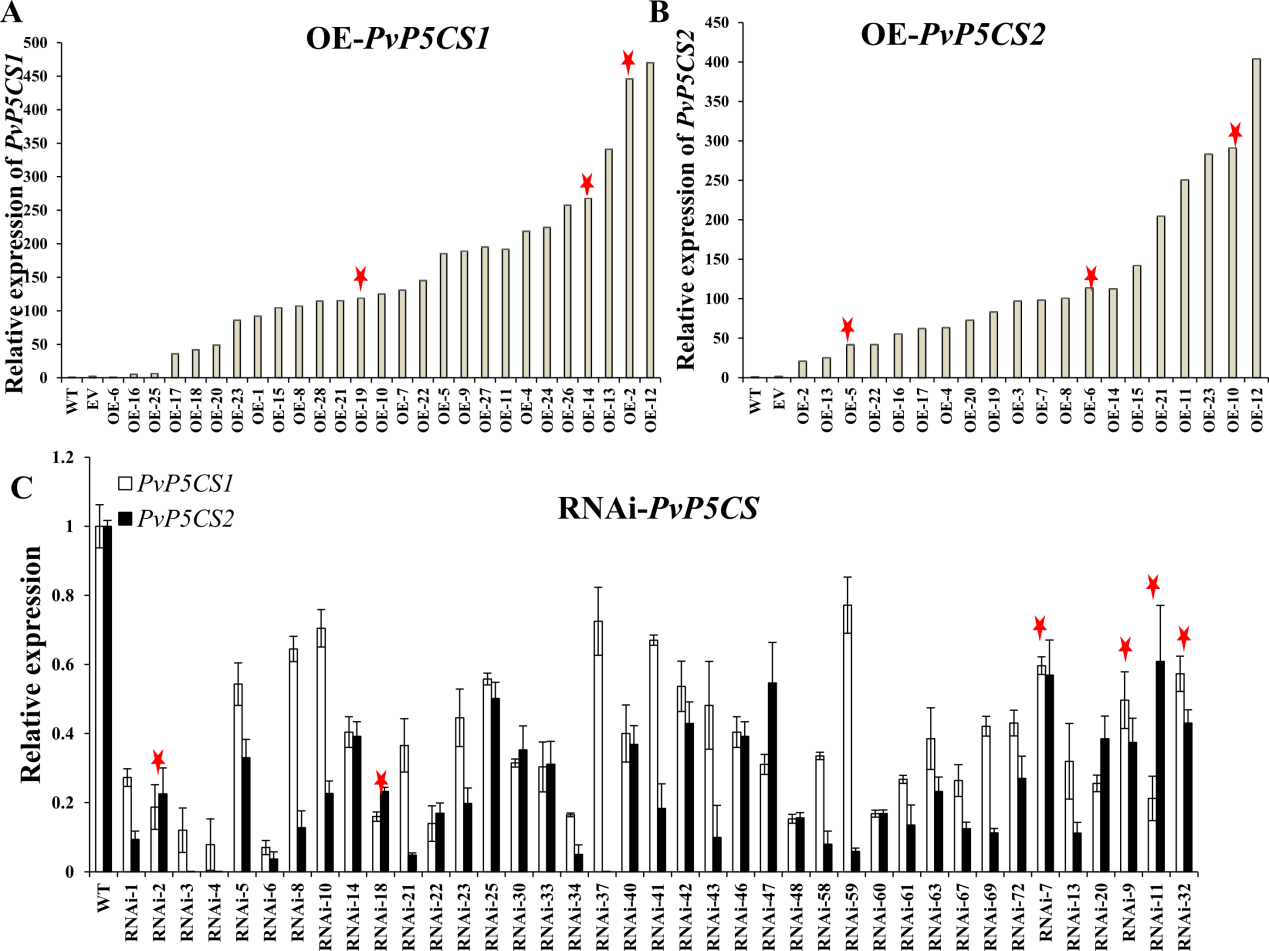


Figure S3. Relative expression levels of *PvP5CS1* and *PvP5CS2* genes. Relative expression levels of *PvP5CS1* in *PvP5CS1 OE* transgenic plants (A). Relative expression levels of *PvP5CS2* in *PvP5CS2 OE* transgenic plants (B). Relative expression levels of *PvP5CS1* and *PvP5CS2* in *PvP5CS* *RNAi* transgenic plants (C). Value are mean ± SE (*n* = 3). The selected OE and RNAi transgenic lines used for the experiment were labeled with the stars in the figure.


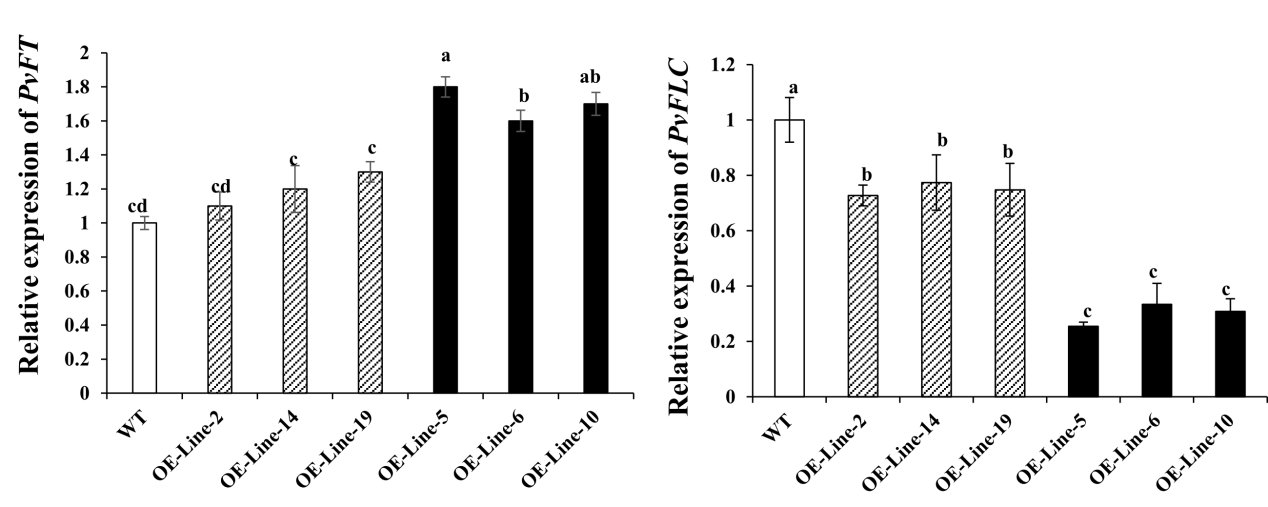


Figure S4. Relative expression levels of *PvFT* and *PvFLC* genes. Data are mean values of three biological repeat, and significance of treatments was tested at the *P* < 0.05 level (one way ANOVA, Dunnett’s test).


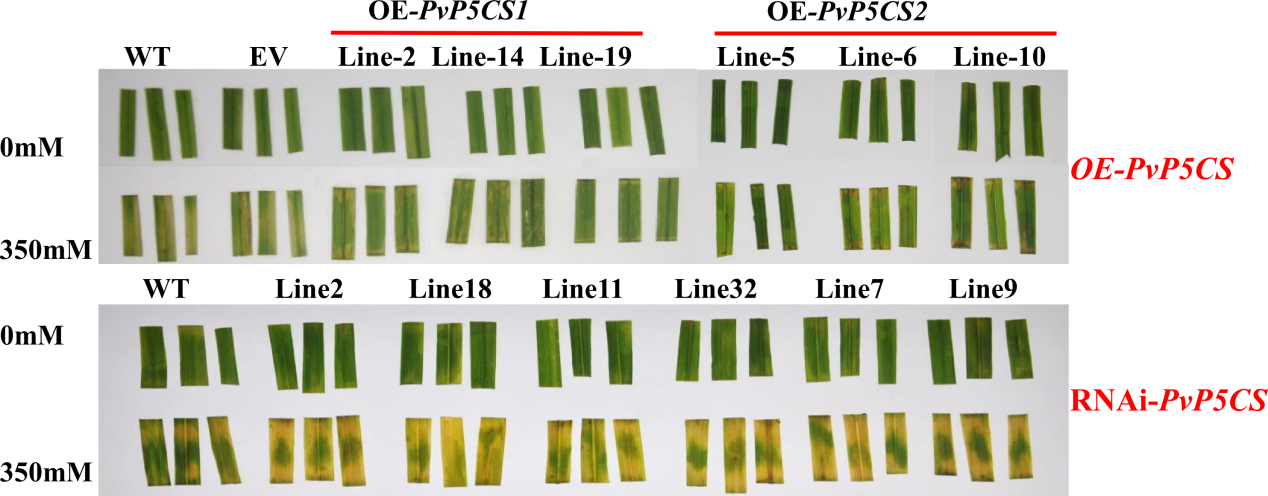


Figure S5. Preliminary evaluation of salt tolerance of *PvP5CS OE* and *RNAi* transgenic lines.


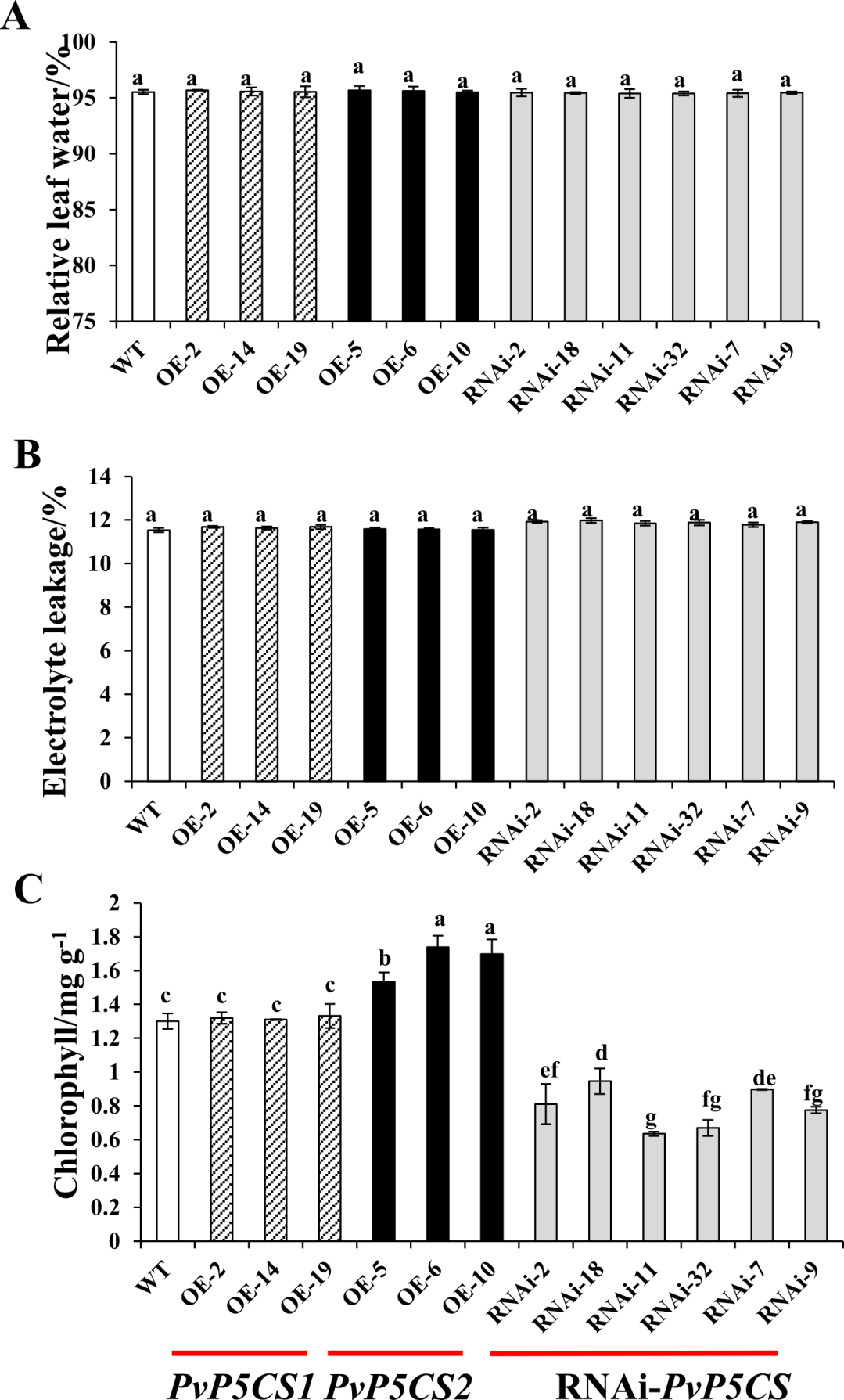


Figure S6. Relative leaf water (A), electrolyte leakage (B) and chlorophyll (C) content in *PvP5CS OE* and *RNAi* transgenic plants under 0 mM NaCl. Data are mean values of three biological repeat, and significance of treatments was tested at the *P* < 0.05 level (one way ANOVA, Dunnett’s test).


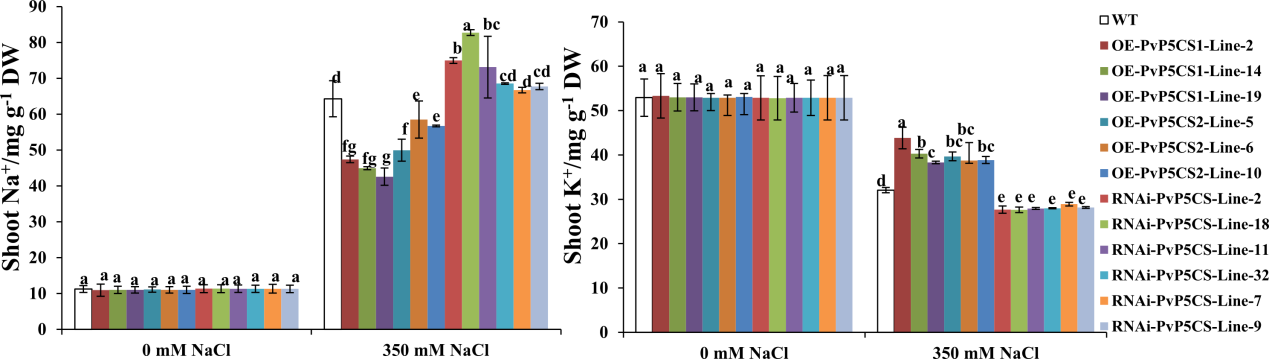


Figure S7. Na^+^ and K^+^ uptake in shoots under normal and salt stress conditions.

Table S1. Primers used in this study

| Name | Sequence（5’-3’） | Purpose |
| --- | --- | --- |
| PvP5CS1  (Pavir.J02344.1)  PvP5CS1  PvP5CS2  (Pavir.J06546.1)  PvP5CS2  PvP5CS1  PvP5CS2  PvCAT  (Pavir.J03636.1)  PvSOD  (Pavir.Ib01670.1)  PvUBIQUITIN1  (Pavir.FL955474.1)  PvSAMDC  (Pavir.Ab02264.1)  PvSPDS  (Pavir.Ba02363.1)  PvSPMS  (Pavir.Ab01042.1)  PvPAO  (Pavir.J39070.1)  Ubi1301 vector | F:ATGGGGAGGGGAGGGATCGG  R:TCATTGCAAAGGAAGATCCCTGTGG  F:GGGGTACC ATGGGGAGGGGAGGGATCGG  R:CGAGCTCTCATTGCAAAGGAAGATCCCTGTGG  F:CCATGGCGACCGCGGACC  R:TGAATCATCGATCAGGCTCAAACAC  F:CGGGATCCCCATGGCGACCGCGGACC  R:GGGGTACCTGAATCATCGATCAGGCTCAAACAC  F:GTGACAAGGGAGTGGTTTACA  R:AACCCGCTTTGACGGTATT  F:CTTCGAAATCCACTCCTCTCTC  R:CCCGTCACCTATCCAGTATTT  F:CATGCTGAGAAGGTTCCTATCC  R:ACCAGCCTGCTTGAAGTTATT  F:TGTCAACTGGACCGCATTAC  R:AGTTAGCAACACCATCATCTCC  F:CAGCGAGGGCTCAATAATTCCA  R:TCTGGCGGACTACAATATCCA  F:CCGCTTGCTGCTGTTAAATAC R:TTCAGGAGGGCAACTTCATC  F:TGATTGTCGATTCCTCTGATCC  R:CAACTCCACCTGGACGTAAA  F:GGGAAGTCACCATACCAAGAAG  R:ACGCGCATTCATCCTTATCA  F:GCAACTTCCTCACCGACTTTA  R:CATCCGCTCGGTCAATTATCT  F: TGATGGCCCTGCCTTCATACGCT  R: TATTGCCAAATGTTTGAACG | Gene cloning  Overexpression vector construction  Gene cloning  Overexpression vector construction  Quantitative expression  Quantitative expression  Quantitative expression  Quantitative expression  Quantitative expression  Quantitative expression  Quantitative expression  Quantitative expression  Quantitative expression  PCR identification |

Table S2. The characterization of growth and development in overexpressing transgenic and WT plants

|  |  | Plant height | Tillers | Internode length | Internode diameter | Leaf length |
| --- | --- | --- | --- | --- | --- | --- |
| WT |  | 76.33±2.31c | 79.11±3.61c | 12.61±0.61d | 2.34±0.20d | 43.17±0.38d |
| OE-PvP5CS1 | Line-2 | 109.17±8.97ab | 104.33±2.52ab | 16.37±0.64b | 4.08±0.16a | 63.67±3.75b |
|  | Line-14 | 101.27±1.53b | 101.31±2.65ab | 18.07±0.059a | 3.68±0.18b | 67.42±2.76ab |
|  | Line-19 | 103.63±0.58b | 107.42±6.08a | 16.27±0.67b | 3.73±0.26b | 68.67±3.02a |
| OE-PvP5CS2 | Line-5 | 102.33±3.21b | 97.61±2.65b | 15.07±0.60c | 3.68±0.16b | 67.71±2.41ab |
|  | Line-6 | 116.39±9.02a | 96.67±2.31b | 15.03±0.65c | 3.67±0.16b | 52.57±3.51c |
|  | Line-10 | 101.67±6.51b | 95.33±2.52b | 16.37±1.21b | 3.15±0.17c | 56.53±2.65c |

Internode length and internode diameter (internode 3), plant height, leaf blade length and tillers were measured in overexpressing transgenic and WT switchgrass when plants were 5 months old. Each line had three biological replicates, and three tillers were measured in each biological replicate. Value is mean ± SE (*n* = 3) in tiller number, and the other value are mean ± SE (*n* = 9). The significance of treatments was tested at the *P* < 0.05 level (one way ANOVA, Dunnett’s test).
